# Transcriptome Analysis of *Paeonia ostii* ‘Fengdan’ Seeds Uncovers Starch and Sucrose Metabolism Conferring High Yield Under Brassinosteroid Treatment

**DOI:** 10.3390/genes16121424

**Published:** 2025-11-29

**Authors:** Shixi Yu, Ruixue Xiao, Xiaopeng Li, Renjie Li, Chengwei Song, Yuying Li, Jingyi Zhao, Xiaogai Hou

**Affiliations:** College of Agriculture, Henan University of Science and Technology, Luoyang 471023, China

**Keywords:** transcriptomics, seed, yield, brassinosteroids, starch and sucrose metabolism

## Abstract

**Background**: Tree peony (*Paeonia section Moutan* DC.) seeds, renowned for their rich content of unsaturated fatty acids, hold significant medicinal and nutritional value. Despite their potential, their yield is relatively low, which constrains economic gains and health benefits. Brassinosteroids (BRs), emerging as potent plant growth regulators, have demonstrated efficacy in boosting plant productivity. **Methods**: This study employed *Paeonia ostii* ‘Fengdan’ and administered either water (control group, CKs) or 0.05 mg/L BRs through foliar application during the seed enrichment and conversion phase to assess the effects of BR treatment on seed yield. **Results**: The BR treatment notably enhanced the protein, starch, and sugar content and yield of *P. ostii* ‘Fengdan’ seeds, surpassing those of the CKs. Transcriptome profiling identified a plethora of differentially expressed genes in *P. ostii* ‘Fengdan’ in response to BR treatment, which are implicated in biological processes associated with nutrient reservoir activity. KEGG analysis highlighted the pivotal role of starch and sucrose metabolism in the BR treatment response. WGCNA revealed key gene expression modules that correlate with physiological traits in *P. ostii* ‘Fengdan’. Furthermore, the quantitative real-time PCR (qRT-PCR) validation of key genes in this pathway revealed that BR treatment enhances yield through a dual mechanism: accelerating the seed-filling process and optimizing developmental timing for earlier maturation. **Conclusions**: Collectively, these findings offer inaugural comprehensive genomic resources delineating the transcriptional regulatory mechanisms of BRs in *P. ostii* ‘Fengdan’ seeds.

## 1. Introduction

Tree peony, a member of the Paeoniaceae family and the Moutan DC. genus, is a perennial woody shrub with extremely high ornamental, medicinal, and oil-bearing value. Traditionally, its seeds were primarily used for breeding. In recent years, attention has been increasingly paid to tree peonies as an oil crop of significant economic importance due to their seeds’ rich oil content, ranging from 24.0% to 37.8% [1]. Not only do tree peony seeds have a high oil content, but they are also rich in unsaturated fatty acids (UFAs), including alpha-linolenic acid (ALA), linoleic acid, and oleic acid, as noted by Refs. [1,2], accounting for approximately 90% of the total fatty acid content. Notably, the proportion of ALA is considerably high (~45%) [1]. ALA has been linked to a variety of health benefits, such as a reduction in blood pressure, the inhibition of platelet aggregation, and a decreased risk of cancer [3,4]. Oil tree peony is increasingly becoming an indispensable woody oil crop. Currently, the cultivation area of oil tree peony spans about 129,000 hectares, yielding 530,000 tons of seed oil annually [5]. However, the industry for oil-producing tree peonies is still chiefly hindered by limited seed yield. While extensive research has been conducted to enhance the UFA content in seeds, information regarding the molecular mechanisms regulating seed yield remains limited. Therefore, understanding and improving seed yield are research priorities. *P. ostii* ‘Fengdan’ was selected as the target species for this study due to its prominent representativeness and benchmark quality. It is the most widely cultivated cultivar for oil production in China, with its planting area accounting for over 70% of the total oil-bearing tree peony acreage. Furthermore, it serves as an industry benchmark, boasting a high seed oil content (37–42%) and an exceptionally high proportion of alpha-linolenic acid (>45%). These attributes make it an ideal model for investigating the regulatory mechanisms of yield and providing insights with broad applicability.

Among the various contributing factors, grain weight stands out as one of the most thoroughly researched traits due to its remarkable stability and heritability across fluctuating environmental conditions. Grain weight is determined by the size of the grain and the rate of filling, with genetic pathways such as proteasome degradation, G protein signaling, and plant hormone signaling primarily associated with the control of grain size [6]. The distribution of carbohydrates governs grain filling, thereby influencing grain weight and crop yield [7].

Brassinosteroids (BRs) play a multitude of roles in regulating important agronomic traits in crops, including plant architecture, panicle and raceme morphology, and grain size and shape [8]. Generally, mutants with defects in BR signaling or biosynthesis exhibit erect leaves, reduced plant height, and smaller seeds. In contrast, genotypes with enhanced BR signaling tend to have larger leaf angles and seeds. Consequently, BRs significantly impact the final yield of crop production [9]. For instance, the rice *dwarf4-1* mutant, which has a defect in BR biosynthesis, exhibits an ideal plant architecture with erect leaves. This architecture reduces shading and lodging, leading to increased grain yield per unit area under high-density planting conditions [10]. Similarly, a slight reduction in the expression of the rice BR receptor gene BR *INSENSITIVE1 (BIN1)* leads to erect leaves and increased grain yield without changes in grain size [11]. BRs primarily enhance plant yield by altering plant metabolism, such as by participating in the regulation of carbohydrate flux [12]. In *Arabidopsis*, the BR-responsive gene *EXORDIUM-LIKE1* is activated under carbon starvation, pointing to a regulatory axis that couples brassinosteroid (BR) signaling with growth control when carbohydrate supply is scarce [13]. Consistent with this, plants carrying the bri1 *Y831F* lesion in the BR receptor accumulate more starch and sucrose and several more amino acids than wild-type controls [14]. Further support emerges from the brassinosteroid–light–sugar1 triple mutant: in addition to exhibiting de-etiolation, dwarfism, compact rosettes, and short roots typical of BR depletion, it displays hypersensitivity to exogenous sugars that can be reversed by spraying BRs [15]. A similar metabolic shift occurs in tomato; fruits of the *dx* mutant, which lacks a functional *CYP85A1/DWARF* BR biosynthetic enzyme, contain less starch and soluble sugar but higher concentrations of free amino acids [16]. Therefore, increasing plant yield through the precise manipulation of BR signaling is feasible. However, the molecular mechanisms underlying the regulation of yield formation by exogenous BRs in *P. ostii* ‘Fengdan’ remain unknown.

RNA sequencing (RNA-seq) facilitates the rapid and efficient identification of genes, particularly those that are functionally important under a variety of physiological conditions or during different developmental stages. In this study, we employed RNA-Seq to conduct a comparative analysis of *P. ostii* ‘Fengdan’ seeds at the seed inclusion enrichment and conversion stage following BR treatment, aiming to uncover genes associated with *P. ostii* ‘Fengdan’ yield. From six tree peony seeds sampled at three developmental stages, 85 214 transcripts were assembled; most genes encoding starch and sucrose metabolism were successfully captured. This annotated transcriptome and its expression atlas provide a foundational resource for dissecting the pathways that govern starch biosynthesis. This research may offer novel insights for the application of genetic engineering to enhance the yield of *P. ostii* ‘Fengdan’.

## 2. Materials and Methods

### 2.1. Plant Material

Plants of *P. ostii* ‘Fengdan’, aged ten years, were grown in the experimental plantation of Henan University of Science and Technology, Luoyang, China (112°36′19.65″ E, 34°39′55.43″ N). A randomized complete block design was adopted, with six independent sub-plots (three for control and three for brassinosteroid treatment). A 2 m protection row surrounded the plot area. On 1 June 2017, during the rapid pod and seed growth phase, whole-plant foliar sprays of either distilled water (CK) or 0.05 mg/L brassinosteroid (BR) were applied at 07:00–09:00 until runoff, using separate sprayers to avoid cross-contamination. Seeds were harvested at 15, 30, and 45 days after treatment (DAT; 16 June, 1 July, and 16 July 2017), immediately flash-frozen in liquid nitrogen, and stored at −80 °C until analysis.

### 2.2. Trait Measurements

Estimating grain yield per acre involves calculating the average grain weight per plant and then extrapolating this to the entire acreage. This is performed by first determining the average weight of grains from a sample of individual plants. Subsequently, the plant density within an acre is determined to find out how many plants are present. Finally, the total grain yield per acre is calculated by multiplying the average grain weight per plant by the number of plants in that acre, providing an estimate of the overall grain production.

Grain starch and soluble sugars were quantified with their respective Megazyme kits as per the manufacturer’s protocol. The protein content of each sample was assessed using a previously reported method [17]. Each treatment should have at least 3 biological replicates.

### 2.3. RNA Extraction, cDNA Library Construction, High-Throughput Sequencing, Raw Data Processing, mappingRNA Extraction, Library Preparation, Sequencing, and Bioinformatic Analysis

Total RNA was isolated from seeds collected at three developmental stages with the RNAprep Pure Plant Kit (Tiangen Biotech, Beijing, China) and subsequently purified using Dynabeads^®^ Oligo (dT) 25 (Life Technologies, Carlsbad, CA, USA). RNA integrity and concentration were evaluated on an Agilent 2100 Bioanalyzer (Agilent Technologies, Santa Clara, CA, USA). For library construction, mRNA was first enriched with Oligo (dT) magnetic beads and then reverse-transcribed into cDNA using N6 random primers. After library quality assessment on the Agilent 2100 Bioanalyzer, single-end sequencing (50 bp reads) was performed on the BGISEQ-500 platform. The resulting raw reads were filtered to remove low-quality sequences (those with >10% N bases or >50% bases with Q < 5). The clean reads were subsequently aligned to the tree peony reference transcriptome (NCBI TSA database, BioSample: SRS1180651) using Bowtie2.

### 2.4. Quantification of Gene Expression, Gene Ontology, and KEGG Pathway Enrichment Analysis

Gene expression quantification was performed using RSEM29 with Fragments Per Kilobase of transcript per Million mapped reads (FPKM) as the normalized metric. Pairwise correlations were computed from FPKM matrices, and Euclidean distances were used to cluster transcript-level expression profiles. Condition specificity was modeled with the statistical frameworks of Robinson & Oshlack [18] and Yu et al. [19]. Genes were declared stage-specific if they exhibited ≥ 5-fold up-regulation (FPKM) and a *p*-value ≤ 0.001 relative to the other time points.

Differentially expressed genes (DEGs) were screened with the *NOISeq* algorithm, adopting a minimum fold change ≥ 2 and a divergence probability ≥ 0.8, with the CK sample serving as the reference control. Hierarchical clustering was executed with the TIGR Multi Experiment Viewer (MeV 4.9.0).

All DEGs were functionally annotated against the Gene Ontology (GO) database (http://www.geneontology.org, accessed on 15 June 2025), and enrichment analysis was performed by comparing the observed gene counts per term with the genomic background. Significance was assessed with a hypergeometric test followed by Bonferroni correction; terms with *p* ≤ 0.05 (corrected) were considered significantly enriched. For pathway-level interpretation, DEGs were likewise mapped to the KEGG database, and pathways retaining a Bonferroni-adjusted *p* ≤ 0.05 were declared significantly enriched.

### 2.5. Weighted Gene Co-Expression Network Analysis

A weighted gene co-expression network analysis was performed using the WGCNA R package (v1.72-1) on raw read counts normalized with the regularized log transformation method from DESeq2 (v1.38.3). After filtering out genes with zero expression or no variance, the blockwiseModules function was used to automatically construct the network and identify co-expression modules. A signed adjacency matrix was built from a similarity matrix of Pearson correlations, using a soft-thresholding power of β = 26. The Topological Overlap Measure (TOM) was then calculated to assess network connectivity. Modules were identified by performing average linkage hierarchical clustering on the topological overlap dissimilarity matrix (DistTOM = 1 − TOM). Highly similar modules were subsequently merged using a dynamic tree cut algorithm with a mergeCutHeight of 0.25.

### 2.6. RT-qPCR Analysis of Genes Involved in Starch and Sucrose Metabolic Pathways

Total RNA was extracted from seeds at three developmental stages as detailed earlier. One microgram of RNA was used to synthesize cDNA for qRT-PCR with the All-in-one First-Strand cDNA Synthesis SuperMix from TransGen, Beijing, China, following the kit’s protocol. Genomic DNA contamination was eliminated using DNase and gDNA Remover components of the kit. Primers for genes implicated in starch and sucrose metabolism (*6-FEH*, *OLE9*, *TPPJ*, *TPS9*) were designed using Primer 5.0 software(Table A1). qRT-PCR was conducted with Top Green qPCR SuperMix from TransGen, China, on a Light Cycler 480II Real-Time PCR System from Roche, Mannheim, Germany, with conditions of 95 °C for 15 min, then 35 cycles of 95 °C for 10 s and 60 °C for 20 s. Each gene target was assayed in six replicates, with the ubiquitin gene serving as a reference. Relative gene expression was determined using the 2^−ΔΔCt^ method.

## 3. Results

### 3.1. Impact of Exogenous Brassinosteroids on Seed Yield and Content of P. ostii ‘Fengdan’

**Figure 1:**
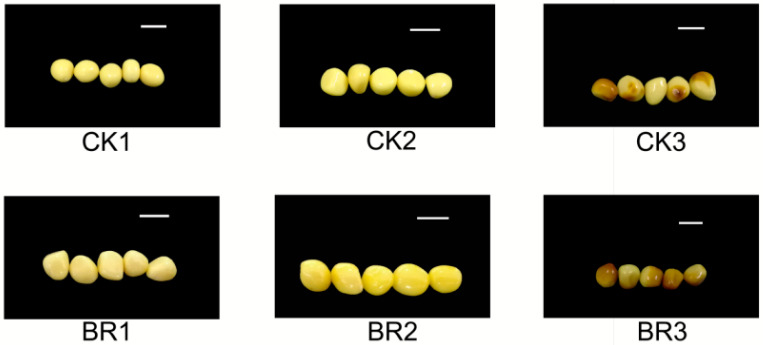
The development status of *P. ostii* seeds across three growth stages. Bar = 1 cm.

**Figure 2:**
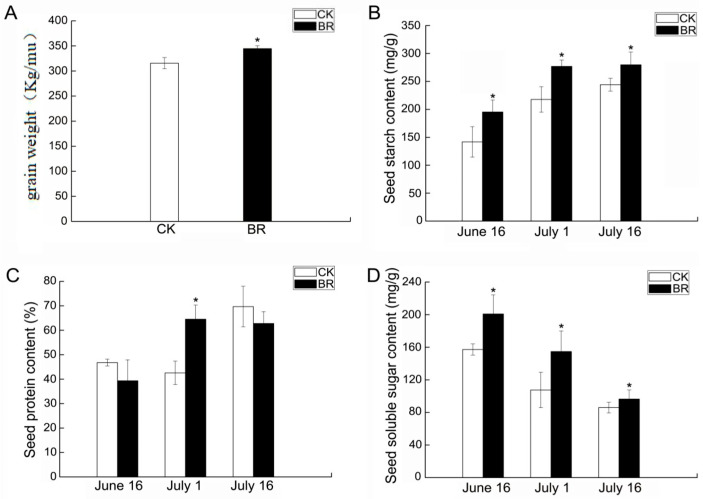
Impact of BRs on yield and grain quality of *P. ostii* ‘Fengdan’ at different developmental stages. (**A**) Grain weight; (**B**) starch content; (**C**) protein content; (**D**) soluble sugar content. Values are mean ± SEM, n = 3. Asterisks indicate significant difference between BRs and CK at *p* < 0.05 (Student’s *t*-test).

Brassinosteroids (BRs) can modulate numerous agronomic traits of significance, including seed weight, seed carbohydrate content, and protein content [17]. To evaluate the impact of BRs on these agronomic traits, we assessed the yield per acre, protein, starch, and soluble sugar content of *P. ostii* ‘Fengdan’ seeds. An increase in seed size was observed in *P. ostii* ‘Fengdan’ following exogenous brassinosteroid (BR) treatment (Figure 1), which was associated with a 9.24% increase in per-acre yield relative to the control (Figure 2). These findings suggest that BR application may enhance the productivity of *P. ostii* ‘Fengdan’. Exogenous BRs also showed varying degrees of enhancement on the protein, starch, and soluble sugar content of *P. ostii* ‘Fengdan’ seeds (Figure 1). As the development of *P. ostii* ‘Fengdan’ progresses, the protein and starch contents tend to increase gradually, while the soluble sugar content tends to decrease. Soluble sugars are progressively converted into starch during seed development [17]. These results confirm that exogenous BR treatment increased the yield and starch and protein content of *P. ostii* ‘Fengdan’ seeds compared with the control groups.

### 3.2. RNA-Seq Analysis of BR-Treated P. ostii ‘Fengdan’

**Figure 3:**
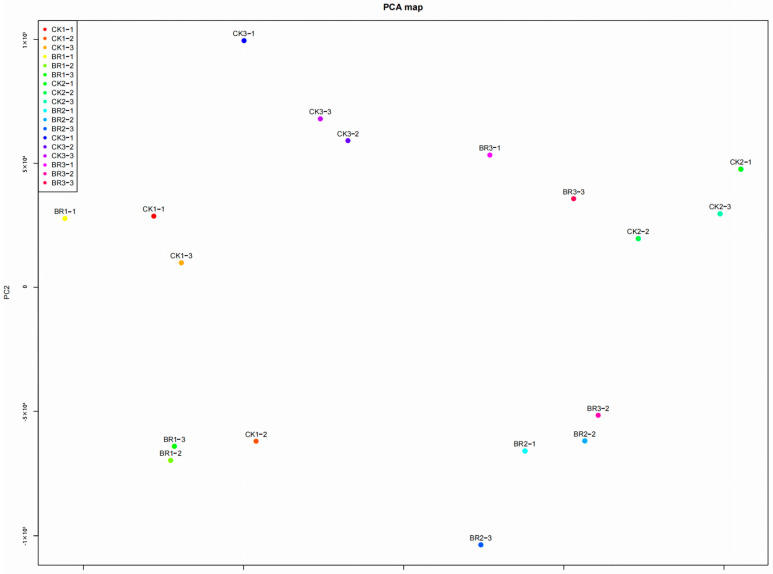
The impact of BRs on the yield and grain quality of *P. ostii* ‘Fengdan’ at different stages.

**Figure 4:**
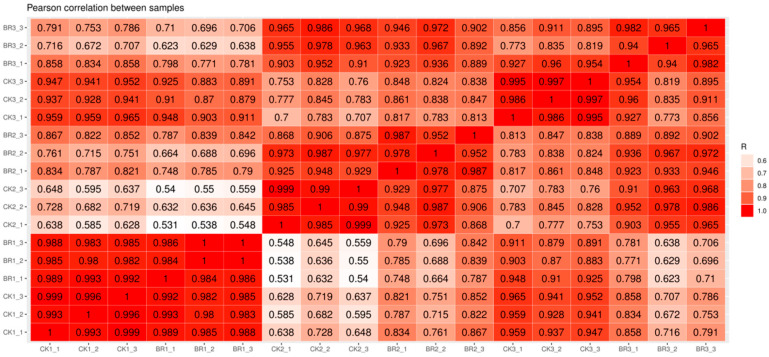
Correlation of RNA-seq data among different treatments.

To assess the impact of BR treatment on gene expression, fresh seed tissues were collected from both BR-treated and untreated *P. ostii* ‘Fengdan’ plants at the seed inclusion enrichment and conversion stage (15, 30, 45 days after treatment, DAT) for RNA sequencing. Consequently, eighteen libraries from seeds at three developmental stages were sequenced, providing a comprehensive transcriptome profile of *P. ostii* ‘Fengdan’ seeds. Sequencing produced an average of 72.1 million raw reads per sample; after filtering poly-N and low-quality sequences, 68.5 million clean reads remained, representing 95.09% of the original data (Table 1). A total of 182.99 Gb of clean data was generated, with an average of 10.166 Gb per sample (Table 1). In total, 85,214 transcripts were obtained from the transcriptome of six tree peony seeds across three developmental stages (Table 2). Seed samples were collected from three tree peonies at each time point, with each tree serving as a biological replicate. The correlation values calculated from the FPKM values of each sample indicated that the three samples collected at each time point had a correlation value of at least 0.94, suggesting a high degree of uniformity among the biological replicates (Figure 3). The separation between the CK and BR treatments was also evident in the corresponding heatmaps (Figure 4), indicating significant differences in gene expression between the two groups.

**Figure 5:**
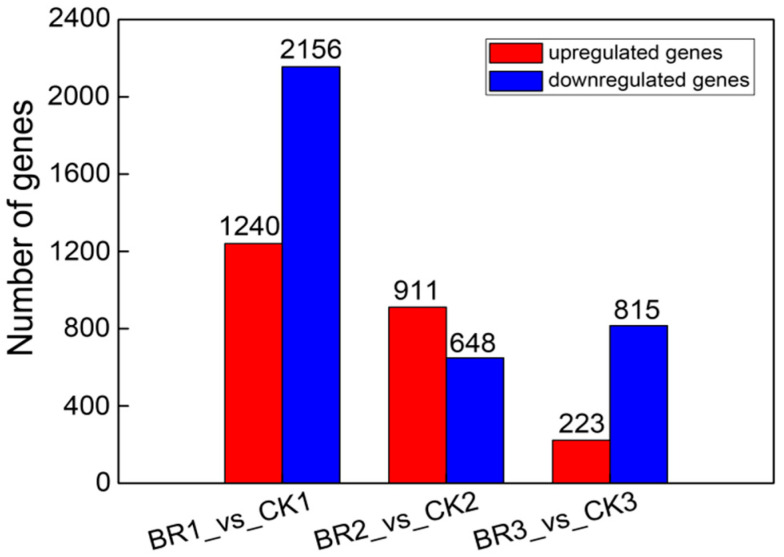
Expression changes in up-regulated and down-regulated genes.

**Figure 6:**
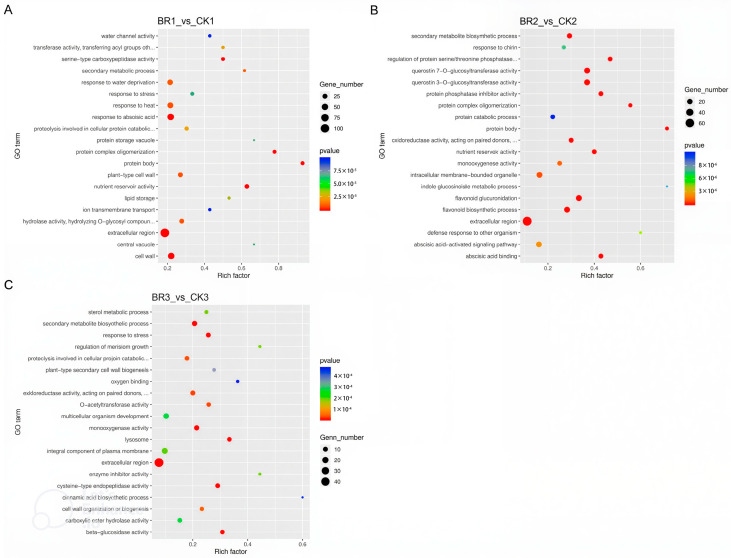
Significantly enriched GO analysis of DEGs in *P. ostii* ‘Fengdan’ seeds from different development periods. (**A**–**C**) ict the GO enrichment analysis of BR1_vs_CK1, BR2_vs_CK2, and BR3_vs_CK3, respectively.

RNA-seq data was analyzed to reveal the trend in the gene expression change in response to BR treatment in *P. ostii* ‘Fengdan’. After three comparisons with the CK samples, differentially expressed genes (DEGs) were identified and subjected to Gene Ontology (GO) functional annotation. At 15 DAT, a total of 3396 DEGs were detected in BR treatment groups compared to the untreated control, including 1240 up-regulated genes and 2156 down-regulated genes. At 30 DAT, a total of 1559 DEGs were detected in BR treatment groups compared to the untreated control, including 911 up-regulated genes and 648 down-regulated genes. At 45 DAT, a total of 1038 DEGs were detected in BR treatment groups compared to the untreated control, including 223 up-regulated genes and 815 down-regulated genes (Figure 5). In the comparisons between the BR treatment group and the CK group across three developmental stages, nutrient reservoir activity and lipid storage were significantly enriched in the 15 DAT samples, while nutrient reservoir activity was significantly enriched in the 30 DAT samples (Figure 6). This enrichment pattern aligns with the observed trends in starch, protein, and soluble sugar content within the seeds (Figure 2). From 15 DAT to 45 DAT, there is a gradual transformation of soluble sugars into macromolecules such as starch and a concurrent increase in protein content, indicating the progressive accumulation of nutrients. Collectively, the GO enrichment analysis of DEGs confirms the outcome of BR treatment in enhancing the accumulation of starch and protein during the development of *P. ostii* ‘Fengdan’ seeds.

**Figure 7:**
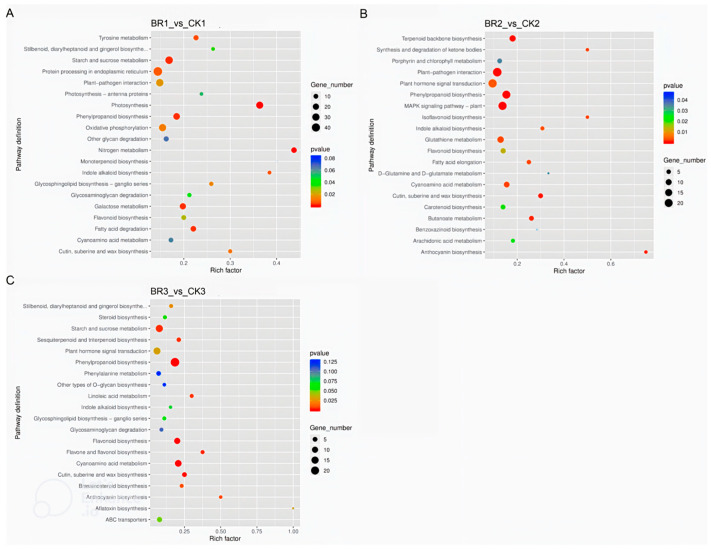
Significantly enriched KEGG pathways of DEGs in *P. ostii* ‘Fengdan’ seeds from different development periods. (**A**–**C**) depict the KEGG pathway enrichment analysis of BR1_vs_CK1, BR2_vs_CK2, and BR3_vs_CK3, respectively.

**Figure 8:**
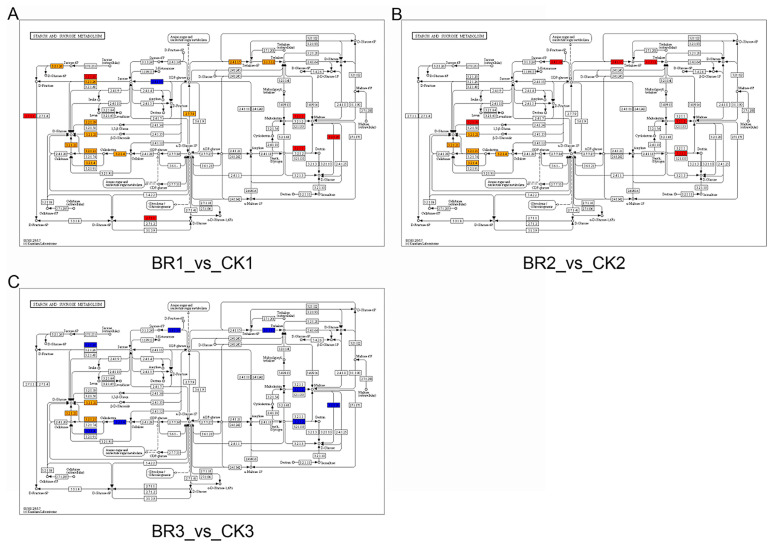
KEGG pathway analysis illustrating the molecular response of *P. ostii* ‘Fengdan’ to BR treatment. (**A**–**C**) depict the sbi00500 pathway of BR1_vs._CK1, BR2_vs._CK2, and BR3_vs._CK3, respectively (Red: *p* < 0.01; orange: *p* ≈ 0.01–0.05; blue: *p* > 0.05; grey: not enriched).

To assess how BR treatment reshapes seed development, we mapped differentially expressed genes to Kyoto Encyclopedia of Genes and Genomes (KEGG) pathways and screened for routes significantly enriched in *P. ostii* ‘Fengdan’. Prior to the peak accumulation of seed starch and protein content (45 DAT), all seed comparisons revealed that the BR treatment group showed significant enrichment in pathways such as flavonoid biosynthesis, starch and sucrose metabolism, fatty acid degradation, and fatty acid elongation compared to the CK group (Figure 7 and Figure 8). Consistent with the GO enrichment results, the KEGG pathway analysis of the DEGs indicated that BR treatment promotes starch and protein biosynthesis during *P. ostii* ‘Fengdan’ seed development.

### 3.3. Gene Expression Network Related to BR Response Identified by WGCNA

**Figure 9:**
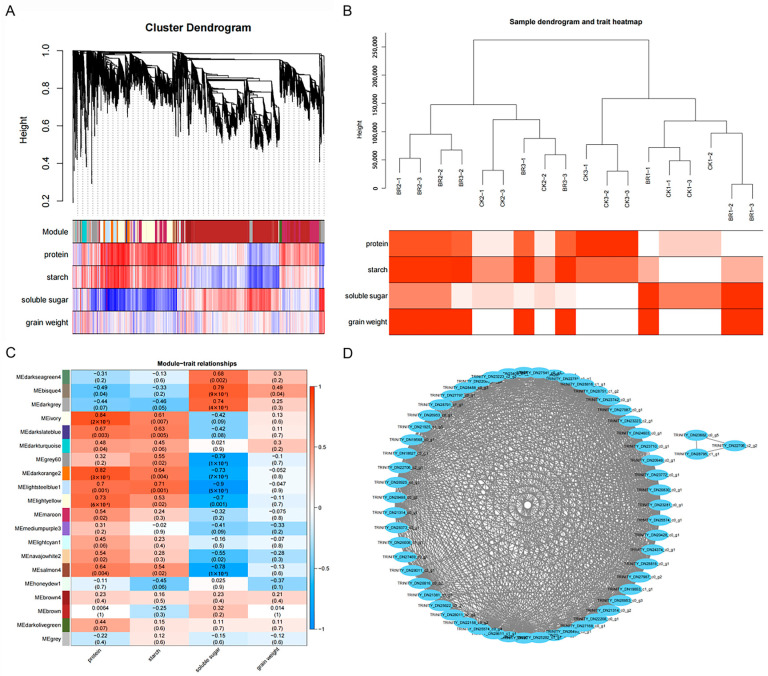
A WGCNA of the *P. ostii* ‘Fengdan’ seeds’ response to BR treatment. (**A**) A hierarchical clustering dendrogram of genes. The upper section depicts the phylogenetic relationships, and the lower section represents the correlation of gene expression with their respective modules; redder hues indicate stronger positive correlations, while bluer shades suggest stronger negative correlations. (**B**) A heatmap of the clustering patterns and associated characteristics for both treatments. (**C**) A heatmap of the Pearson correlations between module eigengenes and stress conditions, with 20 distinct modules identified, each color-coded for clarity. (**D**) The gene interaction network within the midnightblue module.

To further explore the gene expression network associated with the response to BRs, we conducted a weighted gene co-expression network analysis (WGCNA) using the expression data from both CKs and BR-treated samples. This analysis identified a total of 20 gene expression network modules, each containing between 37 and 4220 co-expressed member genes (Table 3). The representative co-expression patterns, referred to as module eigengenes, were then correlated with four previously measured physiological traits. Notably, nearly all modules exhibited significant associations with at least one of the traits (Figure 9C).

Grain weight and soluble sugar, starch, and protein content are important to consider when measuring *P. ostii* ‘Fengdan’ yield. There was a total of 1 module significantly correlated with grain weight, 10 with protein content, 7 with starch content, and 9 with soluble sugar content. The MEbisque4 module with 74 genes showed the most significant correlation with grain weight. The MEdarkorange2, MElightsteelblue1, MElightyellow, and MEsalmon4 modules showed the most significant correlation with soluble sugar, starch, and protein content. We used Cytoscape software (v3.9.1) to map the interaction network of genes in the MEbisque4 module (Figure 9D), which showed a complex web of interactions with a high density of connections among genes. The prominent links between these genes may indicate that they are coordinated in critical biological processes.

Enrichment analysis was performed on the genes within the MEbisque4, MEdarkorange2, MElightsteelblue1, MElightyellow, and MEsalmon4 modules, revealing significant enrichment in pathways such as starch and sucrose metabolism, nitrogen metabolism, and Galactose metabolism. The metabolism of starch and sucrose plays a pivotal role in yield formation, primarily by generating a series of sugars as metabolites that promote the synthesis of essential compounds, including proteins, cellulose, and starch [20]. The transport of sucrose and its metabolism towards starch control the production of starch, which is a major determinant of grain yield [21]. Therefore, elucidating the molecular mechanisms that regulate starch and sucrose metabolism processes offers significant opportunities to enhance the yield of *P. ostii* ‘Fengdan’ through biotechnological interventions.

### 3.4. Expression Levels of Key Pathway Genes in P. ostii ‘Fengdan’ Under BR Treatment

To clarify the expression profiles of genes associated with starch and sucrose metabolism during the development of *P. ostii* ‘Fengdan’ seeds—key players in the conversion of sucrose to starch—we initially analyzed the acquired transcriptome data. Specifically, we enriched differentially expressed genes (DEGs) with an FPKM value > 10 in the sucrose-to-starch metabolic pathway (KEGG: 00520) and further identified four genes (*6-FEH*, *TPS9*, *OLE9*, *TPPJ*) that exhibited distinct differential expression patterns. Subsequently, qRT-PCR was employed to determine the expression levels of these four genes across three developmental stages of *P. ostii* ‘Fengdan’ seeds.

**Figure 10:**
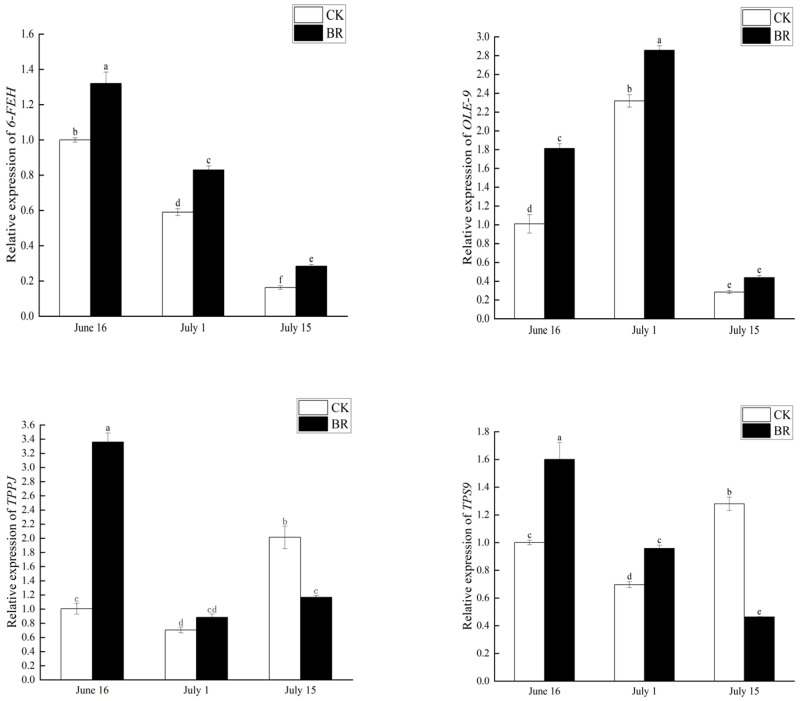
Relative expression levels of starch and sucrose metabolism genes of *P. ostii* ‘Fengdan’ at three time points.

Based on the RNA-seq results, with the PoUBQ gene serving as an endogenous control, the expression of the aforementioned starch and sucrose metabolism-related genes was analyzed. The qRT-PCR data revealed distinct expression dynamics among the four genes: *OLE9* expression first increased and then decreased during seed development, while *6-FEH* exhibited a consistent downward trend in expression throughout the entire developmental period; *TPPJ* showed an expression pattern of an initial decrease followed by a subsequent increase, and *TPPB* displayed treatment-specific characteristics—its expression first decreased and then increased in the control group, whereas a continuous downward trend was maintained in the BR-treated group (Figure 10).

Strikingly, the transcript levels of all four genes were markedly elevated in BR-treated seeds relative to controls at both 15 and 30 d after treatment (15 and 30 DAT; Figure 10). These differential expression trends in the key genes further demonstrate that BR treatment promotes starch and sucrose metabolism by regulating the expression of critical genes in the starch and sucrose metabolic pathway, which aligns with the earlier findings from transcriptome and pathway enrichment analyses in this study.

## 4. Discussion

Tree peony seeds are rich in nutritional and medicinal components, not only offering energy and essential fatty acids to promote cardiovascular health but also possessing medicinal properties such as their ability to clear heat and detoxify, as well as improving sleep quality. Despite their diverse applications, the low yield of tree peony seeds [5] constrains the development of the tree peony oil industry. Therefore, enhancing the yield of tree peony seeds is crucial. Studies have indicated that BRs can increase grain size and, consequently, yield by influencing various physiological processes [22]. Understanding the physiological response to BR treatment and the gene network regulating the molecular response in *P. ostii* ‘Fengdan’ could help identify key genes for improving *P. ostii* ‘Fengdan’ yield. Here we profiled physiological traits and transcriptome dynamics throughout seed development in control versus BR-treated *P. ostii* ‘Fengdan’. The BR treatment group was found to have higher grain weight, starch content, protein content, and soluble sugar content compared to the CK group. Thousands of DEGs were identified in *P. ostii* ‘Fengdan’ in response to BR treatment. DEGs in the BR treatment group showed enrichment in genes related to nutrient reservoir activity, etc. KEGG analysis found that the starch and sucrose metabolism pathway could play an essential role in the response to BR treatment. WGCNA identified important gene expression modules correlated with physiological traits in *P. ostii* ‘Fengdan’. These results enhance our understanding of BR treatment in *P. ostii* ‘Fengdan’ and provides targets for genetic studies.

The metabolism pathways of starch and sucrose are critical determinants of grain yield in plants. Grain yield is primarily contingent upon the distribution of dry matter between photosynthetic source and grain sink tissues. Notably, the ability of grains to transform and store photosynthates is a significant factor limiting yield [21]. Starch, which constitutes up to 70% of the endosperm in grains, largely dictates grain size and weight [22]. In this study, BR treatment significantly enhanced the content of soluble sugars and starch in *P. ostii* ‘Fengdan’ seeds (Figure 2). Therefore, the increase in *P. ostii* ‘Fengdan’ yield due to BR may be driven by enhanced seed filling from increased carbon flux, as demonstrated in the analysis of transgenic rice plants over-expressing *AtDWF4/ZmCYP* [23]. Specifically, rice lines over-expressing *AtDWF4/ZmCYP* show elevated CO_2_ assimilation, higher flag leaf glucose levels, and a more efficient conversion of this glucose into seed starch; the accelerated grain-filling process produces significantly heavier and larger grains [23]. Genes involved in starch and sucrose metabolic reactions have been shown to influence plant architecture, thereby affecting grain yield. The first step in catalyzing the conversion of sucrose to starch is the hydrolysis of sucrose into monosaccharides, glucose/UDP-glucose, and fructose by sucrose synthases, such as *6-FEH*. qRT-PCR analysis revealed that BR treatment significantly elevated the expression of these genes (Figure 10). These monosaccharides are converted into glucose-1-phosphate through a series of reactions, with glucose-1-phosphate being considered the most effective precursor for starch synthesis [24]. Glucose-1-phosphate is converted into ADP-glucose and inorganic phosphate by ADP-glucose pyrophosphorylase (AGPase), providing the substrate for starch biosynthesis [21]. By modulating the expression of key genes in the starch and sucrose metabolism pathways, the starch content and yield of *P. ostii* ‘Fengdan’ seeds can be significantly improved, providing an important molecular mechanism for enhancing *P. ostii* ‘Fengdan’ yield.

To further elucidate the molecular mechanisms underlying this enhanced seed filling, we delved into the expression dynamics of four key genes in the starch and sucrose metabolism pathway. qRT-PCR validation provides a more nuanced, temporal view of how BRs orchestrate this process. Initially, during the early enrichment and conversion stages (15 and 30 DAT), BR treatment markedly up-regulated the expression of all four analyzed genes. The elevated expression of *6-FEH* suggests an accelerated hydrolysis of sucrose, providing a robust supply of substrates for downstream biosynthesis [25]. Concurrently, the increased expression of *OLE9* indicates an enhanced capacity for lipid storage, a crucial component of seed reserves. The higher expression levels of *TPPJ* and *TPPB* imply that BR treatment also primes the regulatory networks governing carbon flux and stress response, collectively creating a highly active metabolic state conducive to rapid nutrient accumulation [20]. This gene expression profile corroborates our physiological data, confirming that BRs effectively accelerate the initiation and progression of the seed-filling process [23].

More intriguingly, the expression patterns at the later developmental stage (45 DAT) suggest that BRs may also promote an earlier completion of nutrient accumulation, thereby advancing the maturation timeline. The most compelling evidence lies in the divergent expression of *TPPB*: while it exhibited a continuous decline in the BR-treated group, its expression rebounded in the control group at 45 DAT. This late-stage upsurge in *TPPB* in CK seeds is characteristic of the transition into the desiccation and dormancy phase, where trehalose synthesis is often enhanced to protect cellular structures. Conversely, the sustained down-regulation of *TPPB* in BR-treated seeds implies that they had likely concluded the primary phase of active nutrient accumulation earlier and were progressing more rapidly towards maturity. This temporal advantage allows BR-treated seeds to achieve a higher final content of starch and protein within the same developmental period, ultimately translating into increased yield [1]. Therefore, our study suggests that BRs enhance seed yield not only by boosting the metabolic rate during filling but also by optimizing developmental timing, ensuring a more efficient and timely completion of seed maturation. This dual regulatory effect offers profound insights for biotechnological applications and genetic strategies aimed at improving the productivity of tree peony.

## 5. Conclusions

By comparing physiological and gene expression differences between the control and BR-treated groups, we found that the expression of genes related to nutrient reserve activity was increased in the BR-treated group. KEGG analysis and WGCNA further revealed the key role of starch and sucrose metabolic pathways in response to BR treatment and identified gene expression modules associated with physiological traits. These findings not only enhance our understanding of the role of BR treatment in peonies but also provide potential targets for genetic research, offering important molecular mechanisms and strategies for improving the yield of tree peony seeds. By modulating the expression of key genes in the starch and sucrose metabolic pathways, the starch content and yield of tree peony seeds can be significantly improved, which is of great significance for advancing the development of the tree peony industry.

## Figures and Tables

**Table 1:** Summary of sequencing data of *P. ostii* ‘Fengdan’ seeds and alignment information for clean reads.

Sample	Raw_Reads	Raw_Bases	Valid_Reads	Valid_Bases	Valid%	Q20%	Q30%
CK1_1	69,940,236	9.86 G	67,510,596	9.27 G	96.53	96.82	91.48
CK1_2	78,634,194	11.09 G	74,233,766	10.28 G	94.40	97.20	92.02
CK1_3	59,538,838	8.39 G	57,568,478	7.93 G	96.69	96.80	91.25
BR1_1	62,783,122	8.85 G	60,346,868	8.26 G	96.12	96.39	90.67
BR1_2	80,168,182	11.30 G	77,484,644	10.73 G	96.65	96.81	91.11
BR1_3	74,773,970	10.54 G	72,013,958	9.96 G	96.31	96.69	90.86
CK2_1	73,030,808	10.30 G	70,241,888	9.71 G	96.18	96.83	91.11
CK2_2	62,725,162	8.84 G	60,350,984	8.35 G	96.21	96.81	91.06
CK2_3	71,055,804	10.02 G	68,583,564	9.49 G	96.52	96.88	91.30
BR2_1	80,086,264	11.29 G	76,328,078	10.61 G	95.31	97.05	91.53
BR2_2	75,388,476	10.63 G	72,120,646	10.01 G	95.67	96.88	91.18
BR2_3	76,141,626	10.74 G	73,096,770	10.14 G	96.00	96.83	91.13
CK3_1	85,975,682	12.12 G	78,414,912	10.74 G	91.21	96.64	90.93
CK3_2	80,145,924	11.30 G	74,536,402	10.27 G	93.00	96.70	90.89
CK3_3	71,487,958	10.08 G	65,966,458	9.06 G	92.28	96.66	90.88
BR3_1	62,049,466	8.75 G	58,120,858	8.01 G	93.67	96.79	91.18
BR3_2	76,144,758	10.74 G	72,021,480	10.00 G	94.58	97.07	91.59
BR3_3	57,815,302	8.15 G	54,521,084	7.53 G	94.30	96.77	91.10

**Table 2:** Overview of Trinity assembly results.

Index	All	GC%	Min Length	Median Length	Max Length	Total Assembled Bases	N50
Transcript	85,214	41.01	201	691.00	16,336	83,855,058	1472
Gene	29,262	41.66	201	837.00	16,336	32,760,180	1661

**Table 3:** WGCNA modules and their corresponding gene counts.

Module	Gene Number
bisque4	74
brown	4220
brown4	183
darkgrey	298
darkolivegreen	107
darkorange2	371
darkseagreen4	37
darkslateblue	69
darkturquoise	175
grey	365
grey60	218
honeydew1	130
ivory	80
lightcyan1	81
lightsteelblue1	438
lightyellow	1381
maroon	1457
mediumpurple3	90
navajowhite2	63
salmon4	163

## Data Availability

The original contributions presented in this study are included in the article. Further inquiries can be directed to the corresponding author.

## Appendix A

**Table A1:** qRT-PCR primer sequences.

Transcript_Id	Forward Primer (5′ → 3′)	Reverse Primer (5′ → 3′)
TRINITY_DN23567_c1_g4	GGCTTGCAAGAATACACCGCA	AAGGGGCTCCACGTTCACAA
TRINITY_DN19592_c1_g1	CGAAGTTCGCGGGTCAATGG	ACACCTTGACCTTCCCACCC
TRINITY_DN23401_c0_g1	GGTGGCACCCGGTGGTTATA	TCAGGGCTGATGGGTGACAA
TRINITY_DN23738_c0_g5	AGATTCTGCCCGTGGGTGTT	GCTCCTGCCGTTGCTGTAAG
